# Traffic Density-Related Black Carbon Distribution: Impact of Wind in a Basin Town

**DOI:** 10.3390/ijerph18126490

**Published:** 2021-06-16

**Authors:** Borut Jereb, Brigita Gajšek, Gregor Šipek, Špela Kovše, Matevz Obrecht

**Affiliations:** Faculty of Logistics, University of Maribor, Mariborska cesta 7, 3000 Celje, Slovenia; borut.jereb@um.si (B.J.); gregor.sipek@student.um.si (G.Š.); spela.kovse@student.um.si (Š.K.)

**Keywords:** black carbon, traffic pollution, traffic density, air pollution, wind conditions, basin city

## Abstract

Black carbon is one of the riskiest particle matter pollutants that is harmful to human health. Although it has been increasingly investigated, factors that depend on black carbon distribution and concentration are still insufficiently researched. Variables, such as traffic density, wind speeds, and ground levels can lead to substantial variations of black carbon concentrations and potential exposure, which is even riskier for people living in less-airy sites. Therefore, this paper “fills the gaps” by studying black carbon distribution variations, concentrations, and oscillations, with special emphasis on traffic density and road segments, at multiple locations, in a small city located in a basin, with frequent temperature inversions and infrequent low wind speeds. As wind speed has a significant impact on black carbon concentration trends, it is critical to present how low wind speeds influence black carbon dispersion in a basin city, and how black carbon is dependent on traffic density. Our results revealed that when the wind reached speeds of 1 ms^−1^, black carbon concentrations actually increased. In lengthy wind periods, when wind speeds reached 2 or 3 ms^−1^, black carbon concentrations decreased during rush hour and in the time of severe winter biomass burning. By observing the results, it could be concluded that black carbon persists longer in higher altitudes than near ground level. Black carbon concentration oscillations were also seen as more pronounced on main roads with higher traffic density. The more the traffic decreases and becomes steady, the more black carbon concentrations oscillate.

## 1. Introduction

Air pollution and global warming have been thoroughly researched in recent years, particularly as the harmful effects of human activities on Earth are increasing, in addition to the warming of the atmosphere. The human population faces a higher risk of climate-related liability, in regards to health, water supply, food security, and decreased economic growth [1].

Greenhouse gasses (GHGs) are one of the main global warming agents, but their warming effects are not alike. Presumably, the most known polluter is carbon dioxide (CO_2_). According to the United Framework Convention on Climate Change (UNFCCC), the effects of different GHGs accountable for warming of the atmosphere “can be converted into the Carbon Dioxide (CO_2_) equivalent by using the Global Warming Potential (WGP) of each GHG” [2]. In addition to GHGs, Earth’s atmosphere is directly and indirectly altered by atmospheric aerosols, with a shorter lifespan than GHGs [3]. One atmospheric aerosol is black carbon (BC), also known as “soot”, and sometimes defined as a GHG, since it causes climate change [4]. These particles can absorb and scatter solar radiation in the atmosphere. They act as cloud condensation nuclei, and some of them (e.g., BC) may even have a warming impact [5]. Furthermore, aerosols diminish visibility, have a significant impact on air quality, and can have negative consequences on human health, as stated by Becerril-Valle et al. [5] and Nordeide Kuiper et al. [6], especially for exposed elderly individuals [7], children [4], newborns, and fetuses during pregnancy [8]. Reducing particulate matter (PM) on sites where PM concentrations and population density are priorities can provide new insights on this topic. 

According to Nazeer Husssain et al. [9], BC is one of “the most powerful climate forcing agents”. Safai et al. [10] state that BC is a more impactful emission than methane while being second only to CO_2_. Most BC aerosols in the atmosphere originate from human-made activities [11], where incomplete combustion of fossil fuels and biomass is present [12]. Pey et al. [13] specified pollution sources in more detail, stating that the main ones are transport, industry, burning of biomass, and secondary sources. 

Even though BC is defined as a short-lived climate pollutant [14], it is possible to observe its distribution, because BC emission apportionment depends on several factors of anthropogenic or natural origins; therefore, various aspects need to be taken into consideration. Firstly, the amount of fumes containing BC varies in time; hence, concentrations of BC are not the same throughout the day, week, or even year [15]. For example, BC concentrations tend to build up in winter while decreasing in warmer parts of the year [16]. Many studies [17] explain that seasonal fluctuations are mostly a consequence of increased biomass burning in cooler months of the year when the need for heating the domiciles arises or even haze season [18]. Secondly, BC concentration apportionment is dependent on concentrations at the source or at distance from the source [19]. In addition, wind significantly affects BC concentrations and apportionment with its speed and direction. Baldwin et al. [20] proved that increased wind speed helps lower BC concentrations near the ground level. Moreover, all types of precipitation have a notable effect on BC concentrations. As Kucbel et al. [21] stated, the relationship between water and BC accumulation is dictated by the hydrophobic character of freshly emitted particles and precipitation intensity. 

As stated, traffic is one of the most influential contaminants of anthropogenic origin, such as BC emissions from vehicles, more specifically from diesel engines, posing up to 43% of all PM_2.5_ particles. PM_2.5_ refers to atmospheric particulate matter (PM) with a diameter of less than 2.5 micrometers, a part of which is BC particles [15]. In urban areas, BC concentrations are mostly influenced by traffic density, where traffic rush hours should be stressed. According to a paper by Liu et al. [22], traffic peaks are usually divided into two parts: morning and afternoon rush hour. Furthermore, the weekend effect can be detected in urban areas. Reduced BC concentrations are typical of this occurrence and result from lower traffic density on weekends [3]. BC concentrations from traffic are also considerably dependent on the presence or absence of road canyons. These anthropogenic constructs usually restrain maximum concentrations of air pollutants, according Wagner and Schäfer [23]. Various natural and anthropogenic barriers, such as buildings, sound walls, or trees alongside traffic routes, substantially impact the transmission of BC particles [6,24,25].

The most important anthropogenic sources of primary particulate matter (PM) in ambient air in Europe are (i) exhaust and non-exhaust emissions from road traffic, and (ii) combustion of solid biomass [26]. There is convincing evidence that PM, almost regardless of the source, has detrimental health effects [27]. Excessive human exposure to BC can be a health concern [28], even in places with less air pollution [29]. Studies indicate that short- and long-term exposure to BC can lead to respiratory [30,31] and cardiovascular [32,33] diseases and increased mortality [34,35]. The World Health Organization estimated that, in 2016, ambient air pollution caused 4.2 million premature deaths worldwide, in both cities and rural areas [36,37]. The authors of [38] found evidence of an association among BC, a marker of traffic pollution, and lung functions in women. Risk factors contributing to lung function are of interest, given that COPD is indicative of the loss of lung function [39]. Public health implications are substantial, because COPD was projected to be the fourth leading cause of death worldwide by 2020 [27]. This list of studies showing the negative impact of BC on health is long and trustworthy. Improving air quality is a considerable, but not intractable, challenge [40], especially if the BC phenomenon is to be intensively investigated. 

To date, studies have measured traffic-related air pollution and its sources [14,41], including BC, and investigated health impacts (i.e., Becerril-Valle et al. [5]), differences in the impacts on population segments [4,7], transportation modes, and influential factors, such as fires, winter heating, season, or daytime [14,16,17,18]. Recent studies also investigated BC distribution in cities, in regards to barriers, green belts, and traffic density [6,22,24,25,42]. However, there is a lack of studies related to specific environments (e.g., basin towns, in regards to traffic density and road segments). In particular, weather (e.g., wind) and road segments encased by tall buildings could influence the appearance of after-effects, which can be seen in the suppression of exhaust fumes and dusty particles reducing the quality of air near the ground level. Many studies show that aerosol distribution and exposure can vary significantly, and is considerably underestimated (as well as under-researched) for specific environments and altitudes (levels) [6,42].

The present study aimed to characterize variations of traffic density-related black carbon (BC) concentrations for different neighborhoods with different characteristics. In doing so, real-time monitoring in spring and winter, in a small town located in a basin, with high smog occurrence temperature inversion, and low wind occurrence, were carried out. The goal was to define distribution and occurrence in specific, less-airy sites, which might be—due to higher BC concentration—even more risky to human health. Our main motivation for the study was to investigate if BC concentrations in a basin town were significantly different in winter and spring than in more windy areas. With this in mind, our research was conducted to measure and study the distribution of BC emitting from traffic in the municipality of Celje (MOC), with measuring points on different altitudes and ground levels, and different distances from roads, with dense traffic. It was carried out to compare measurements in winter and spring, differing among BC concentrations, from biomass burning in winter, and transport-related BC pollution in relation to wind speeds. This manuscript is structured as follows: Section 2 specifies the materials and methods utilized, describes environmental specifics and measurement locations, and measurements of BC and traffic density. Section 3 reports the results of traffic density and BC concentrations in spring and winter under different circumstances. Section 4 presents the discussion and Section 5 the concluding remarks.

## 2. Materials and Methods

This study was designed to measure BC concentrations and occurrences at different measuring points/stations at specific sites in a small town, located in a basin, with respect to traffic density and selected other factors (e.g., wind).

### 2.1. Site Description

**Figure 1:**
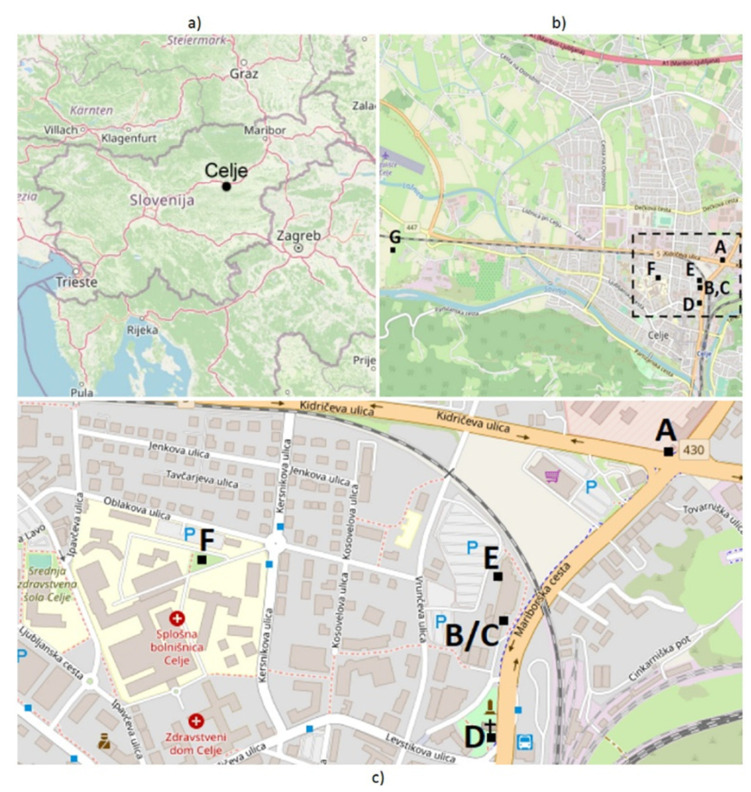
(**a**) Location in the region, (**b**) BC measuring points on the map of Celje with surroundings from OpenStreetMap, and (**c**) BC measuring points on the map of Celje center, from OpenStreetMap.

Celje (in MOC) is known as one of the coldest cities in Slovenia, as it is situated in a basin where the temperature inversion is common (Figure 1a). This phenomenon is most frequent in the winter, but it occurs in the summer as well. It is a consequence of cooler and thicker air sinking into the basin, where it lingers for long periods, as it cannot escape from the concavity. The inversion above Celje is usually 60 to 150 m deep, expanding up to 32–128 km^2^ of the area. In the winter, the inversion begins to scatter at around 11:00 a.m. to 1:00 p.m., while in the summer the process of discarding begins a bit earlier, around 8:00 to 10:00 a.m. [43]. The Celje basin is one of the least airy regions in Slovenia, and, as already stated, inversion is common, which leads to excessive pollution. As hard particles in the atmosphere are cooler than the surrounding air, they represent a condensation nucleus that captivates the moisture. This natural phenomenon leads to “polluted fog,” also called smog [44].

Celje is situated against the corridor 5 (RFC 5), the main traffic axis of Koper port’s logistics center. It also connects the two biggest cities of Slovenia–Ljubljana and Maribor, and further expands to Austria and Hungary. The mentioned traffic corridor surrounds Celje in the north, where it collides with the city. In addition, Celje is intersected by two ring roads—the conjunction of Kidričeva road with a traffic hub in Medlog, where the west motorway junction is laid, and Mariborska road with the northern motorway junction and intersection to Aškerčeva street [45].

Even though the roads of Celje are mainly open and airy, where exhaust fumes cannot linger for longer periods, there are some sections where “road canyons” are present. These are the road segments encased by tall buildings, and the after-effect can be seen in the suppression of exhaust fumes and dusty particles that reduce air quality near the ground level. These road sections in Celje can be identified on the Mariborska road in the direction N–S, between Kidričeva and Levstikova street, and between Levstikova and Cankarjeva street.

In MOC, the main sources of pollution are industry, technological processes and industrial boiler houses, road traffic, boiler rooms for heating of domiciles and preparation of sanitary water, and residential fireboxes. The environmental report of MOC states that a detailed source analysis in 2011 was initiated because of the surplus in permitted daily values of PM_10_ particles in the air at the MOC area. PM_10_ describes inhalable particles with diameters that are generally 10 micrometers or smaller. The mentioned analysis showed that up to 31% of dust particles originate from traffic and industry. Biomass burning contributes 24% of the particles, 17% of which are of secondary origin brought around by local air masses from other regions. Suspension of road dust contributes to pollution with 9% of particles and 19% of particles undefined [46].

### 2.2. Measurements Description

Data were gathered in several periods in the winter and spring of 2017 and 2018. Spring measurements were carried out from 14 March to 14 April, 2017, and from 20 to 29 April, 2017. Winter measurements continued throughout December 2017 and from 1 to 25 January, 2018. These measurements provided insight into BC behavior to enable the identification of BC distribution pattern. As stated, measurements took place in Slovenia’s third-largest city, Celje, in MOC, as it is the most representative basin city with low winds in this geographical area. Five fixed measuring points were determined (A, B, C, D, and E) as presented in Figure 1b,c, where aethalometers were set up for gathering data, for the expressed purpose of this research. These locations were selected to show potential differences among BC concentrations at different distances from main roads (Mariborska and Kidričeva; see Figure 1c) and local roads. Data on pollution levels in the city background were provided by the measuring station Celje Hospital (Location F, Figure 1c), while wind speed and its direction, as well as data on precipitation (rainfall and snowfall), were collected from the measuring station AMP Gaji (Location G, Figure 1b). Both measuring stations are included in the state measuring network and provide data on general air pollution and weather conditions in MOC. Measurement locations are presented in Table 1.

Measurements of BC concentrations were carried out in the winter and spring to divide traffic-related emissions (those that occurred in the spring, after the end of the heating season) from emissions combined from traffic and biomass burning (occurred in the winter–in heating season). When comparing these measurements, it was possible to divide traffic-related and biomass burning-related emissions. However, it should be noted that cars need to be warmed up longer in the winter than in the summer, and demand more time to set the operating temperature; thus, winter BC concentrations could be slightly higher.

#### 2.2.1. Fixed Measuring Point A

Measuring point A was at the intersection of Mariborska road and Kidričeva street, which is Celje’s largest crossroad. Eight driving lanes with heavy traffic flow, with up to 20,657 diesel-engine vehicles in an average day intersect here. The numbers are even higher in the rush hour. The aethalometer was set up on a lamppost, approximately 3 m above ground level. Measurements at location A were conducted in the spring as well as in the winter.

#### 2.2.2. Fixed Measuring Point B–A Reference Point

This measuring spot was situated on the ground floor of the building with dimensions of 20 × 110 m. The inlet air in aethalometer was placed in the corner of the building’s ground floor at the height of 3 m. This side of the building faces the main traffic route Mariborska road, which is about 20 m distant and ascends right from the road underpass that causes “a canyon effect.” Measurements at point B were conducted all the time, in spring and in winter, as this fixed measuring point was a reference point for comparison of measurements. Even though measurement results from any two measuring points could be compared, with the help of the reference point, the ratio between any measuring point and point B could be calculated and then used in the research of BC distribution.

#### 2.2.3. Fixed Measuring Point C

Fixed measuring point C was placed right above point B. The inlet air in the aethalometer was installed on the fourth floor of the building, at the height of 18 m above ground level. This installation site was chosen to observe changes in BC concentrations with augmentation of height. Measurements here were conducted only in the spring.

#### 2.2.4. Fixed Measuring Point D

The fourth measuring point was set up in the spring. The aethalometer was attached to a lamppost right by the roadside, opposite Celje’s main bus station, at the height of approximately 3 m and at a distance of about 2 m from the Mariborska road.

#### 2.2.5. Fixed Measuring Point E

As one of the goals of obtaining measurements was to determine how physical obstacles affect BC distribution, this measuring spot was located at ground floor (as well as point B), but at the back of the same building. It is important to note that, even though measurements were conducted at the back of the building, and the instrument was not facing the roadside, vehicles still slightly affected the results. This is because a large public car park is situated at the back of the building, where cars and other vehicles mainly drive across the parking lot in the morning and at the end of the workday, since the majority of people park their cars at this site while they are at work. Moreover, an 80 m distant, and a less important street with low traffic density, may slightly affect the measurement results at this measuring point. Measurements here were also conducted in the springtime only.

#### 2.2.6. Measuring Station F

The fifth measuring station in Celje, labeled Location F, is withdrawn from the traffic. It is situated in the hospital area where traffic is not as dense as on main roads or intersections. At the nearest road, approximately 12,000 motorized vehicles pass by this measuring point daily, while on Mariborska road, the number of vehicles is almost double—about 23,000 vehicles per day. Here, at Celje Hospital, sulfur dioxide (SO_2_), nitrogen dioxide (NO_2_), carbon monoxide (CO), PM_10_ particle concentrations, and ozone concentrations were measured. Winter measurements in December 2017 and January 2018 were conducted at this spot.

#### 2.2.7. Measuring Station G

The second measuring station, labeled Location G, is AMP Gaji, at a municipal automatized measuring station. It is located in the eastern part of the city near the district heating plant. This station gathered data on sulfur dioxide (SO_2_), nitrogen dioxide (NO_2_), PM_10_ particle concentrations, benzene, and ammonia, as well as wind speed, wind direction, and the amount of precipitation. Data on the wind (and precipitation) for this research were obtained from this weather station.

### 2.3. Aethalometer Measurements

Measurements for observing BC concentrations and distributions in MOC were conducted with several devices of the Aethalometer^®^ Model AE-33, provided by Magee Scientific, Aerosol. This instrument’s basic sampling principle is the same as in older models; the operation and calculations are based on the Ångström exponent. The airflow through the aethalometer enables continuous aerosol particles sampling onto the quartz filter, where particle attenuation is measured at seven different wavelengths, which allows spectral analysis of the data [47]. BC mass concentration was calculated from the change in optical attenuation at 880 nm in the selected time interval [48]. The two-component model considers the aerosol optical absorption coefficient as a sum of biomass burning and fossil fuel combustion fractions, and takes advantage of the difference in the wavelength dependence of absorption. Since fossil fuel and biomass contributions to aerosol absorption feature specific values of the absorption Ångström exponent, it was possible to construct a source specific two-component model [48,49]—BC concentrations originating from traffic (BCtr) and BC concentrations stemming from biomass burning (BCbb) [49]. For this research, the devices were set to 1-min intervals, but because the received data on weather conditions were listed in time intervals of 30 min, all further processing was made at 30-min intervals.

All measurements were conducted in different seasons (winter and spring), meaning, diverse meteorological conditions were encompassed and studied in the research. Moreover, average working days were selected for analysis of the gathered data. These were days during the school year without annual vacations and when public offices had longer working hours.

### 2.4. Traffic Density Measurements

The gathering of data on traffic density was ordered by MOC, more precisely, by a Directorate of the Republic of Slovenia for infrastructure [50]. The data were clustered, based on the numbers, collected via manual and automatic counting of traffic density countywide. Manual counting runs in 15-min intervals on an average working day, between 5:30 a.m. and 9:30 p.m. Data gathered were normalized to get the normalized number of vehicles (NNV). For buses, lightweight and heavy trucks, factors that are multiples of a personal vehicle were selected (separately for each type of vehicle. According to data of the Republic of Slovenia’s statistical office in 2017, 51% of private vehicles ran on gasoline, 47% with diesel engines, and 2% alternative (gas, electric vehicles) [51].

When studying air pollution as a consequence of traffic density, it was assumed that one bus or one truck equaled four diesel-engine personal vehicles. This number stems from the fact that fuel consumption and the level of emitted particles are four times higher than average in buses and trucks than in personal vehicles when accelerating or driving with even speed. It is important to note that fuel consumption for trucks varies according to the amount of freight they transport (0–20 t on average) [52].

## 3. Results

### 3.1. Traffic Characteristics

MOC intersects with numerous vital traffic communications, one of them being Mariborska road, with the largest intersection in town, which was also one of the fixed measuring points (measuring point A).

**Figure 2:**
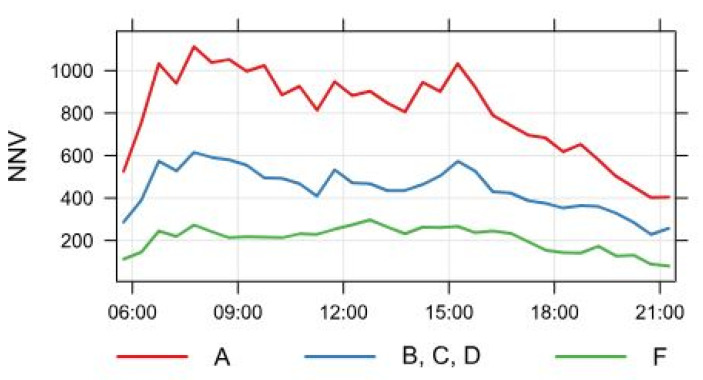
Normalized number of vehicles (NNV) on an average day at measuring points.

Mariborska road represents the main traffic route of MOC and, therefore, has heavy traffic flow. On an average working day, 20,657 diesel-engine vehicles drive through the intersection of location A, of those 16,175 private vehicles, 233 buses, and 4249 freight vehicles. NNV at point A can be seen in Figure 2 and is presented with the red line. Following this line across the graph shows that vehicles at point A are most numerous in the morning rush hour between 6:30 and 8:00 a.m. The graph peaks heavily in that time period because the working class is heading to work. The same happens in the afternoon rush hour between 1:00 and 3:30 p.m. when people leave work. The NNV in this time period reaches its highest values, which start to diminish after 4:00 p.m.

At measuring point B, numbers are slightly lower. The NNV for this measuring spot is shown in Figure 2 and is marked with a blue line. The blue line shows the average number of NNV for measuring points C and D, as the traffic flow is the same for all three measurement areas. Here 12,920 diesel vehicles represent the mean traffic flow in a working day; 10,011 of those are private motorized vehicles, the number of buses is 204, and freight vehicles are numbered at 2705. Daily rush hour was also taken into consideration at point A. Morning rush hour at this spot is again most vivid between 6:00 and 8:00 a.m. when daily commuters are on their way to work and/or school. A slight deviation upwards is seen on the graph (Figure 2) in this time period. This is also the main reason for an increase in the number of private motorized vehicles on the roadside. Because Mariborska road is the busiest traffic route in Celje, it also represents the main bus route. Therefore, an increase in the number of buses in the morning rush hour is evident, especially during the school year. Again, the afternoon rush hour begins at 1:00 p.m. and is most prominent until 3:30 p.m., as most of the working class leave their jobs. In Figure 2, a peak can be distinguished shortly after 1:30 p.m. and is moderately descending until 4:00 p.m. Afterwards, the blue line can be seen plunging faster until 9:00 p.m.

Another measuring spot (point F) was put up at an urban background, where the Slovenian Environment Agency (ARSO) placed a measuring station. The NNV for this spot is marked in Figure 2 with a green line. Here, the traffic flow is significantly lower than at the A or B measuring points, which can be unquestionably seen from the graph. The average number of diesel vehicles in a working day here stands at about 5655, of which the vast majority are private motorized vehicles, numbered at 5286. The number of buses is 93, and the amount of freight vehicles is 276. Here, in the urban background, morning rush hour can be partitioned into two parts—one from 6:00 to 7:00 a.m. and the other between 7:30 and 8:15 a.m. This occurrence is also visible in Figure 2. In the first part, the number of private motorized vehicles rises the most as this is the time when early daily migrations to work occur. In the second period of morning rush hour, the rest of the workers head to work. Moreover, at this time, the number of buses increases, particularly throughout the school year, not only in the morning, but also in the afternoon rush hour. Thus, two timeframes can be taken into consideration. The first one is evident from 10:30 a.m. to 1:00 p.m. In contrast, the second can be observed between 1:45 p.m. and 3:15 p.m. Again, the number of private motorized vehicles is augmented because of the end of the working day, and the same goes for buses, especially during the school year. It is important to note that in the afternoon the number of freight vehicles increases, too. Delivery may be the main reason for that.

### 3.2. Black Carbon Concentrations and Distribution—Lessons Learned

Mean concentrations of measured BC and wind speed obtained from measuring points A, B, C, D, E, and F in the winter and spring, and air temperature obtained from measuring point A only (due to insignificant differences among temperature at different measuring points) are presented in Table 2. In the winter, concentrations for measuring points A and B are higher, while they are significantly lower in the spring. The most significant difference (56.8% decrease in springtime) in average BC concentration values is observed at measuring point B, where traffic flows out from the underpass under the railway. There is a noticeably smaller difference in average BC concentration values at the busiest crossroads with the traffic light at measuring point A—only a small 3.1% decrease in the spring. At measuring point F, with the smallest daily flow of vehicles, we detected the smallest average BC concentration values in the winter observation, 55.7% lower than at measuring point A and 46.6% lower than at measuring points B/C/D. The wind was, on average, 4.9% stronger in the winter. Spring temperatures were, on average, 10% higher than winter temperatures; however, this study did not focus on temperature/emission relationship, but it will be investigated in our future research.

**Figure 3:**
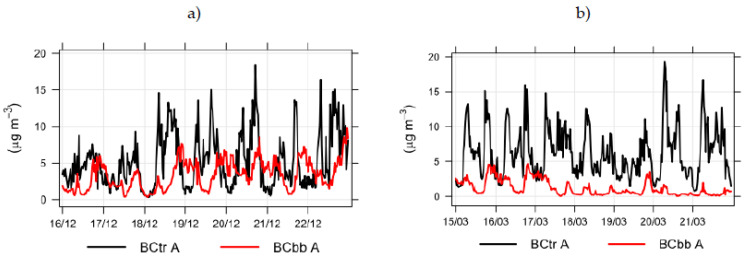
BC concentrations originating from traffic (BCtr) and biomass burning (BCbb) during winter (**a**) and spring (**b**) at measuring point A.

It was assumed that high BC density in colder months occurs mainly due to biomass burning for household heating. To better understand the relationship between BC concentrations originating from traffic (BCtr) and BC concentrations stemming from biomass burning (BCbb), we show this graphically in Figure 3.

In Figure 3, the red line describes BC concentrations originating from biomass burning (BCbb). Presentation of data for point A was selected due to the highest measured traffic density and pollution with BC. Still, BC concentrations stemming from biomass burning were significantly higher in the winter (panel a) than in the spring (panel b), where they resulted mostly from traffic. Concentrations peaked in the evenings and maintained high values until 10:00 p.m. due to housing heat. BC concentrations originating from biomass burning were lowest between 12:00 and 5:00 a.m. This pattern could be seen in the winter and spring. However, concentrations were lower in the spring due to higher outdoor temperatures and the gradual end of the heating season. BC concentrations originating from biomass burning (BCbb) were slightly lower during the weekend than on working days.

**Figure 4:**
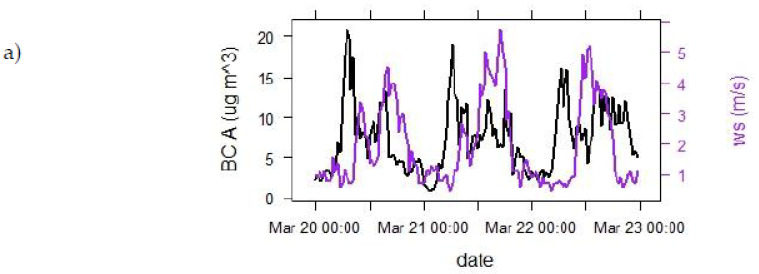

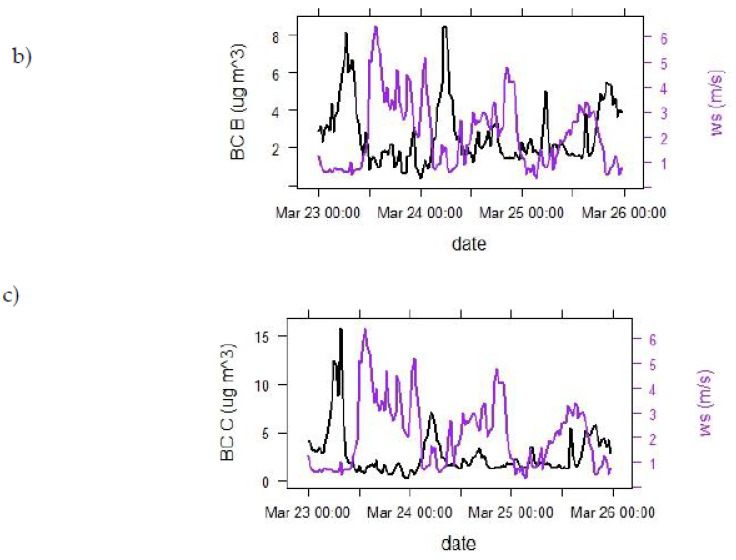
BC concentrations and wind values for measuring point A (graph **a**), measuring point B (graph **b**), and measuring point C (graph **c**).

In Celje, low wind speeds and temperature inversions are quite common. At all measuring points, we observed that BC contraction values decreased with increasing wind speed (as expected). In our case, the wind intensified in the afternoon and significantly decreased BC concentrations during the afternoon traffic peak. The difference between morning and afternoon peak concentrations would otherwise be smaller at low wind speeds. In the calm periods, when there was no wind or when the wind reached speeds up to 1 ms^−1^, the BC concentrations rose. Figure 4 presents BC concentration values compared to wind speed values in the spring for three measurement points (A, B, C).

**Figure 5:**
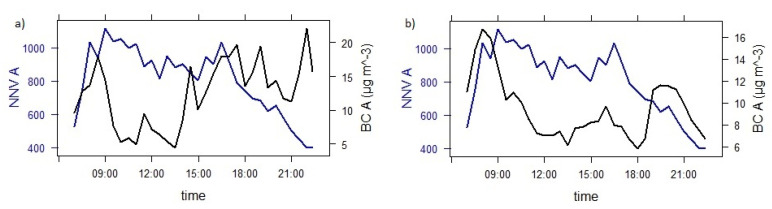
BC concentrations in dependence to normalized number of vehicles (NNV) in winter (**a**) and spring (**b**) time at measuring point A.

The relationship between BC concentrations and the normalized number of vehicles (NNV) in the winter (graph a) and spring (graph b) at measuring point A are presented in Figure 5.

BC concentrations peaked at morning and afternoon rush hours because of larger traffic density, which can be seen in Figure 5. In the evenings of the winter period, the BC concentrations increased further due to the heating of houses. In the spring, the evening increase in BC concentrations is smaller. Graphs for other measurement points also show a similar movement of BC concentrations as a function of NNV.

**Figure 6:**
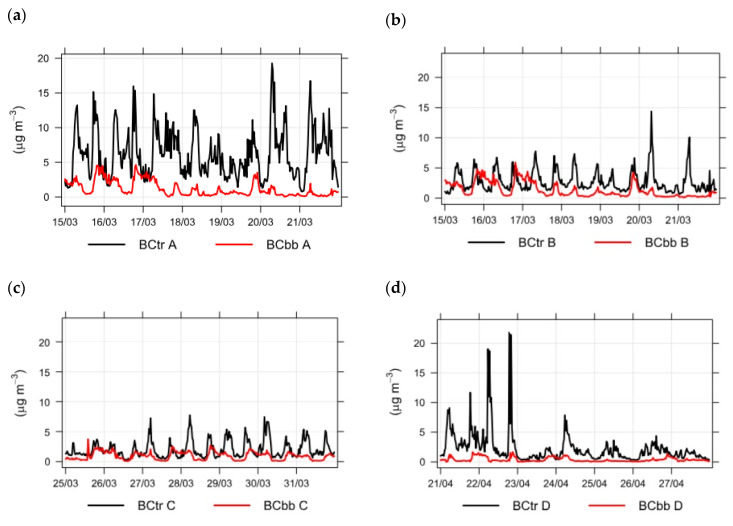
Comparison of springtime BC concentrations at measuring points A (**a**) compared to measuring points B (**b**), C (**c**), and D (**d**).

From Figure 3 and Figure 4, we recognize that decreased traffic flow on weekends is accompanied by smaller BC concentrations. The comparison of BC concentrations at measuring points A, B, C, and D in the spring is presented in Figure 6.

An increase in BC particle density was visible at all measuring points. The concentration was the highest at measuring point A, regardless of winter or springtime (7.48 ± 6.48 µg m^−3^ in winter and 7.25 ± 6.06 µg m^−3^ in spring). The main reason was the location and the role of the crossroad near measuring point A. That crossroad is the largest traffic light intersection in MOC. Vehicles often stop, wait for a short time, and accelerate. Still, spring measurement results were 3% lower in BC concentration than those in the winter. At measuring point B, which was set up in one of the rooms on the ground floor of a four-story building, concentrations were 46.6% lower. This spot was situated 3 m above ground level and 20 m from an area where Mariborska road ascends from the road underpass. Mean BC concentration values in measuring point B reached 6.20 ± 5.07 µg m^−3^ in the winter and 2.68 ± 2.65 µg m^−3^ in the spring. In the warmer spring period, BC concentrations dropped by 56.7%. In spring, measurements were also conducted at measuring point C, which was located on the fourth floor, 20 m above measuring point B. Here, average concentrations of BC were somewhat higher, namely 2.75 ± 2.29 µg m^−3^. The wind speed in that period was 1.91 ± 1.43 ms^−1^, which was not a significant influence factor, but still, by observing the results, it can be concluded that BC persists longer in higher altitudes than it does near ground level. Spring measuring point D was 135 m distant from measuring spot B, and set up 2 m from the roadside where Mariborska road turns into Levstikova street, near the main bus station in Celje. Results of measurements at point D gave the average BC concentrations of 2.50 ± 3.94 µg m^−3^. It is important to emphasize that spot D is in close proximity to another, smaller traffic light crossroad. In this intersection (next to measuring point D), vehicles queue and accelerate just like at point A. The difference is that the crossroads near measuring point A covers a larger area, has more driving lanes, and the traffic flow is much denser. Moreover, measuring point D is at a more open space where wind at a lower speed can more actively affect BC concentrations and BC distribution.

Another measuring point (site E) was set up in spring. It was placed at the back of the same building already bearing measurement points B and C, next to a public car park of MOC. Mean values of BC concentrations in measuring point E fluctuated at 1.67 ± 1.43 µg m^−3^. It is necessary to emphasize that rush hour in this area does not have a significant influence on BC concentrations. At the same time, peaks in particular time periods can be ascribed to the movement of vehicles around the parking lot and to the deliveries.

In the urban city background at measuring site F, winter and spring measurements were conducted. The average winter BC concentration was 3.31 ± 3.25 µg m^−3^, around 25% lower than average BC concentration in measuring point A and almost 50% lower than those in December at the same measuring site. The reason for such a deviation between December and January is a decrease in BC concentrations from biomass burning, as the average temperature in January was 2.2°C higher than in December and, therefore, the need for residential heating decreased. The speed of the wind was also an essential factor. In December, the average wind speed was 1.91 ± 1.86 ms^−1^, while in January, it was higher, 2.04 ± 1.70 ms^−1^.

While conducting the research and discussing the data, a slight downtrend in BC concentrations was noticed when precipitation was present. Even though some authors have described the same occurrence, this research does not have enough data to link rainfall and decrease in BC concentrations with certainty, as, at the time of these measurements, the amount of precipitation was small. Moreover, rain was rare, and only for short periods of time, with a small amount of precipitation. More measurements would be needed to confirm the hypothesis.

## 4. Discussion

This study confirmed insights from previous studies that investigated traffic-related BC pollution, its source, and health impacts [4,5,7,14,41]. The authors mention a lack of studies related to specific environments, such as traffic density and road segment in a basin city. Celje meets all of the characteristics of such a city. It represents an excellent starting point for verifying assumptions and finding new connections between influential factors, consequently enabling better management of PM and BC pollution in populated areas.

The observations took place at three measuring spots (A, B/C/D, F) along significantly differently dense traffic flows. The densest flow of cars was around 20,000 vehicles per day, considerably less than the flows in big cities, at city entrances. In our case, the traffic through the town was still smooth, and we mostly did not notice congestion or long queues. Vehicles on the busiest, most intensive roads, waited at traffic lights from 2 to 6 min during peak hours, otherwise it was under 2 min. On the least busy street, near measuring point F, 25% fewer vehicles were observed than on the busiest street, and only 6% of all trucks were spotted compared to the busiest road. The research findings from the observations correlate with smooth traffic, without congestion, a basin city with less than 38,000 inhabitants, and traffic flow with a focus on smooth traffic, based on a green wave, which could significantly decrease BC concentrations and, thus, decrease the potential impact on human health (especially pedestrians and cyclists).

Although we did not observe any traffic congestions typical of capitals, we detected morning and afternoon rush hours more pronounced on the main transport routes intended for arrival to and departure from the city. The morning peaks are shown as three characteristic peaks, just before 7:00, 8:00, and 9:00 a.m., associated with the start of work or “sliding” working hours. Companies that start operating at 6:00 a.m. are located more on the city outskirts; transportation is regulated by public passenger transport and time-adjusted due to a large number of employees per company traveling on the same route. The type of business activities located in city centers, or parts of cities, affects vehicle flow along individual traffic routes, with opening hours and shift schedules. The afternoon rush hour peaks appear shortly after 2:00 and 3:00 p.m. when employees from large industrial facilities and production companies leave work. We expected a peak after 4:00 p.m., but we did not notice it. This can be associated with the fact that employees from large industries and production companies leave the workplace at the same time; employees from the service sector (e.g., banks, post offices, shopping centers), and others who finish their work at different times in the afternoon, have different lengths of lunch breaks, which disperses the departure times from work. If, in the morning, rush hour peaks are visible at all measuring points—in the afternoon, we noticed them only at the busiest measuring point in a non-traffic street. There is actually no peak, but a slight increase over a longer period of several hours. The BC concentration oscillations are undoubtedly more pronounced along with the busiest routes. The more the traffic decreases and becomes steady, the less the BC concentrations oscillate.

Because the aethalometer, which we used for measurements, enables BC measurements separately for BC concentrations originating from traffic and from biomass burning, we observed fluctuations in these values according to the season [49]. Seasonality of the movement of BC concentrations was confirmed once again, as expected. In the winter, BC concentrations are, on average, higher, while in the spring, they are significantly lower. High BC density in colder months is mainly the consequence of biomass burning, used for heating in buildings. Peak times in BC concentration are, due to household heating on biomass, entirely different from the BC concentration at peak times, due to traffic flows. BC concentrations from biomass burning peak mostly in the evenings, when most urban residents heat their homes. BC concentrations from biomass burning reach the lowest average values between 12:00 and 5:00 a.m.

When dividing traffic-related and biomass-related emissions, it should be noted that cars need to be warmed up longer in the winter than in the summer and demand more time to set operating temperatures, so wintertime BC concentrations could be impacted by internal combustion engines and could be higher, especially close to parking.

This study confirms that wind speed and temperature inversion have a noticeable impact on average wintertime BC concentrations. With this in mind, it was interesting to find out how this, exactly, influences BC dispersion. Our study reveals that, in periods when there was no wind or when the wind reached speeds up to 1 ms^−1^, the BC concentrations rose. In lengthy wind periods, when the wind speeds were up to 2 or 3 ms^−1^, BC concentrations began to drop. Consequently, when windy, BC concentrations during rush hours were lower. The same appears to be true in the case of BC concentrations from biomass burning. Stronger wind lowers the BC concentration at the measuring point. The lesson learned is that—solutions that could potentially decrease BC concentrations (e.g., green wave, smart traffic control, limited speed, reducing waiting times) are of special importance for basin cities. It can be speculated that (some) measures that are more costly or time-intensive could be feasible for implementation in a basin city, but may not be that feasible in, e.g., windy cities, where the potential impact would not be as significant. Whiteman et al. [53] indicate that, along with atmospheric stability, especially low temperatures below 0 °C, snow cover can quadruple PM_2.5_ concentrations. However, snow cover was not investigated in this study due to lack of snow in the year of measurements. Chemically stable CO_2_ and other passive greenhouse gases can help to predict PM_2.5_ variations/projections, as stated in the study performed in similar atmospheric conditions in a valley [54]. Temperatures and weather conditions are therefore seen as significant factors impacting BC concentrations; however, they were not the focus of this study.

Differences in BC concentration independence, with ground distance and altitude, were investigated. Suppression of exhaust fumes and dusty particles, which reduces the quality of air near the ground level, was riskier for pedestrians, cyclists, and motorcyclists but less so for users of public transportation (e.g., bus passengers) as partially investigated by Quang et al. [55]. Surprisingly this study detected higher BC concentrations at higher altitudes (measuring point D). It was expected that 20 m of height difference would actually lower BC concentrations compared to the ground position (measuring point B). In that period, the wind speed was 1.91 ± 1.43 ms^−1^, which was not a significant influencing factor. By observing the results, it can be concluded that BC persists longer in higher altitudes than near ground level.

Other research mentioned the phenomenon of canyoning. This study did not note any impact of canyoning, which we attribute to very low buildings with no skyscrapers. The city is built very evenly, without extreme changes in height and without specific belts, such as green belts or skyscraper belts, which can impact BC concentration and distribution.

This study is a response to research calls that requested more studies be conducted on BC concentration and distribution, in different environments and sites, with a focus on special local conditions that can multiply concentrations and, consequently, impact human health. We join the research efforts in establishing tools for efficient monitoring of PM emissions such as BC, informing the public about potential exposure. Variations in the distribution of traffic density-related BC concentrations, for different neighborhoods with different characteristics, in the spring and winter, show potential for inclusion in national (or even global) schemes to reduce human health-related risks and exposure related to pollution, especially for inhabitants living in basins with a high occurrence of smog and temperature inversion, and low wind speeds.

## 5. Conclusions

This research focused on BC emissions and their dispersion. Certain explorations of BC were made in the global science community, e.g., BC aerosols and their characteristics; trends of BC concentrations; concentrations and dispersion of air pollutants from traffic. However, to our knowledge, studies focused only on BC distribution, and factors of influence were not numerous, especially when dealing with specific environments, such as highly populated basins with low wind speeds. This paper presents our approach to BC particle concentration analysis, with a focus on the distribution of BC particles, factors that affect the apportionment of the particles, and in what way concentrations of BC aerosols are altered.

The number of vehicles in the direct closeness of the measuring points was taken into consideration; therefore, our results abide by real-time traffic density data. This helped us with more precise measurements and accurate comparisons of measured concentrations in different spots across MOC. Another decisive argument of ours is also a reference point (measuring point B). This has enabled us to compare measurements from all survey spots, while the ratio remained unchanged, as it was possible to calculate it.

In the future, BC research could be upgraded with “longer-lasting” measurements. It could also be conducted in different (or more severe) weather conditions, and at other (more numerous) measuring spots, monitoring BC concentrations for different geographical areas, or even neighborhoods with different characteristics (e.g., time, traffic, altitude, weather, etc.). This shows potential for inclusion in national/global schemes to reduce human health-related risks and exposure related to pollution, especially for inhabitants living in basins with a high occurrence of smog and temperature inversion, and low wind speeds.

## Figures and Tables

**Table 1:** Descriptions of measurement locations in Celje.

Location	Location/Site	Elevation/Altitude	Winter Measures	Spring Measures	Location	Measurements	Traffic Density
A	Main crossroads	3 m above ground	X	X	On crossroad of two main roads in Celje	Traffic density, BC	Very high (the highest among all sites
B	Faculty building next to main road	3 m above ground	X	X	10 m to main road (Mariborska)	Traffic density, BC	Very high
C	Faculty building next to main road	18 m above ground		X	10 m to main road (Mariborska)	BC (Traffic density same as point B)	Same as site B
D	Main bus station	3 m above ground		X	On the main road (Mariborska) and bus station	BC (Traffic density same as point B)	Very high
E	Parking west side of faculty building	3 m above ground		X	Distant from main roads	BC	Very low
F	Hospital (ER)	2 m above ground	X		Distant from main roads	Traffic density, BC, SO_2_, NO_2_, CO, PM_10_	Very low
G	Suburban area (AMP Gaji)	3 m above ground	X	X	Distant from main roads	SO_2_, NO_2_, PM_10_, benzene, ammonia, wind speed, wind direction, the amount of precipitation	Low

**Table 2:** Data on average BC concentration values, wind speed, and air temperature at measuring points A, B, C, D, E, and F (point G did not measure BC, only weather conditions).

Site	Winter Measurements (December 2017–January 2018)			Spring Measurements (March–May 2017)		
	BC (µg/m^3^)	WS (m/s)	T (°C)	BC (µg/m^3^)	WS (m/s)	T (°C)
A	7.48 ± 6.48	2.01 ± 1.84	2.91 ± 4.93	7.25 ± 6.06	1.91 ± 1.43	11.70 ± 6.47
B	6.20 ± 5.07			2.68 ± 2.65		
C				2.75 ± 2.29		
D				2.50 ± 3.94		
E				1.67 ± 1.43		
F	3.31 ± 3.25					

## Data Availability

The data presented in this study are available upon request from the corresponding author. The data are not publicly available due to restrictions and potential further publications.
